# Investigation of the phosphorylation of *Bacillus subtilis* LTA synthases by the serine/threonine kinase PrkC

**DOI:** 10.1038/s41598-018-35696-7

**Published:** 2018-11-26

**Authors:** Frédérique Pompeo, Jeanine Rismondo, Angelika Gründling, Anne Galinier

**Affiliations:** 1 Aix Marseille Univ, CNRS, LCB, Marseille, France; 2 Section of Microbiology and MRC Centre for Molecular Bacteriology and Infection, Imperial College London, London, SW72AZ UK

## Abstract

*Bacillus subtilis* possesses four lipoteichoic acid synthases LtaS, YfnI, YvgJ and YqgS involved in the synthesis of cell wall. The crystal structure of the extracellular domain of LtaS revealed a phosphorylated threonine and YfnI was identified in two independent phosphoproteome studies. Here, we show that the four LTA synthases can be phosphorylated *in vitro* by the Ser/Thr kinase PrkC. Phosphorylation neither affects the export/release of YfnI nor its substrate binding. However, we observed that a phosphomimetic form of YfnI was active whereas its phosphoablative form was inactive. The phenotypes of the strains deleted for *prkC* or *prpC* (coding for a phosphatase) are fairly similar to those of the strains producing the phosphoablative or phosphomimetic YfnI proteins. Clear evidence proving that PrkC phosphorylates YfnI *in vivo* is still missing but our data suggest that the activity of all LTA synthases may be regulated by phosphorylation. Nonetheless, their function is non-redundant in cell. Indeed, the deletion of either *ltaS* or *yfnI* gene could restore a normal growth and shape to a Δ*yvcK* mutant strain but this was not the case for *yvgJ* or *yqgS*. The synthesis of cell wall must then be highly regulated to guarantee correct morphogenesis whatever the growth conditions.

## Introduction

The bacterial cell wall is a crucial structure that protects the cell from external stresses or damages and confers shape and resistance to osmotic pressure. Lipoteichoic acid (LTA) is an important component of the cell wall in Gram-positive bacteria, which is linked to the membrane by a lipid anchor. The polymer is composed of a polyglycerolphosphate (poly(GroP)) backbone chain tethered to the membrane by a diglucosyl-diacylglycerol glycolipid^1–3^. LTA has been shown to play an important role in bacterial growth and physiology, cation homeostasis, morphogenesis and cell division^4–7^. Unlike *Staphylococcus aureus* which only has one LTA synthase (LtaS) that produces LTA^8^, four LtaS paralogues LtaS, YfnI, YvgJ and YqgS are present in *Bacillus subtilis*. Three of them are authentic synthases whereas YvgJ acts as a primase that adds the first glycerolphosphate subunit to the lipid anchor^9^. All enzymes are active *in vitro*. LtaS is described as the “house-keeping” enzyme necessary during vegetative growth, YfnI is assumed to be the “stress” enzyme important during cell envelope stresses and YqgS is important during sporulation. They are predicted to have five N-terminal transmembrane helices followed by a large extracellular enzymatic domain that is cleaved just after a conserved AXA motif by type I signal peptidases and released in the culture medium^10,11^. Proteomic studies showed that both extracellular domains of LtaS and YfnI (eLtaS and eYfnI) are found in *B*. *subtilis* late-exponential phase culture supernatants when grown in minimal medium^12^. Interestingly, in the absence of LtaS, YfnI becomes more efficient in LTA production and synthesizes polymers with increased length suggesting that YfnI activity is modulated by LtaS^9^. The four LTA paralogues, that possess a high percentage of sequence similarity, therefore seem to have interdependent activities and a partial functional redundancy in *B*. *subtilis*^9^.

In addition, YfnI and LtaS were found to be phosphorylated. YfnI has been identified as phosphorylated protein in two independent phosphoproteome studies on *B*. *subtilis*^13,14^. Furthermore, in the eLtaS crystal structure the active site Thr297 was found to be modified by a phosphate group^5^. The replacement of this Thr by an Ala residue abolished LtaS activity. The authors suggested that it rather mimics an intermediate state than a phosphorylation by a kinase since this residue is located in a deep pocket but the donor for this phosphate group is unknown and remains an open question. In *B*. *subtilis*, several intracellular Ser/Thr protein kinases (STPK) have been characterized, including PrkC, PrkD and YabT. Among them, PrkC is the kinase most highly expressed during exponential and stationary growth phases^15^ when the LTA synthases are active. PrkC was found to be responsible for the phosphorylation of many proteins involved in different cellular processes including metabolism, cell division and morphogenesis^16–20^ and could be responsible for the phosphorylation of YfnI and LtaS. Interestingly, a link has also been established between one PrkC substrate, the YvcK protein involved in the maintenance of morphogenesis during growth on neoglucogenic carbon sources and the LTA synthase YfnI^21^. More specifically, the *yfnI* gene was identified in a transposon mutagenesis screen looking for Δ*yvcK* suppressive mutations; it was further demonstrated that deletion of *yfnI* in a Δ*yvcK* mutant could restore the growth of this strain on neoglucogenic carbon compounds. It has also been shown that phosphorylation of YvcK by PrkC is involved in *B*. *subtilis* morphogenesis. Namely, overproduction of YvcK could rescue the growth and shape defect of an Δ*mreB* mutant and phosphorylation of YvcK was necessary for this rescue^19^. Considering all the previous data, our hypothesis was that YfnI, and maybe some of the other LTA synthases, could also be regulated by phosphorylation by PrkC.

We therefore investigated the potential phosphorylation of YfnI and its paralogues and looked for its regulatory role in LTA synthesis or in cell morphogenesis in *B*. *subtilis*. We showed that the PrkC protein kinase is able to phosphorylate the four LTA synthases *in vitro*. Regarding YfnI, its phosphorylation neither affected its export across the cell membrane nor its release in the culture medium nor prevented binding to its substrate. However, we observed that its phosphoablative form is inactive *in vivo* whereas its phosphomimetic form is active. Altogether, these results suggest a regulatory role of phosphorylation of YfnI on its activity with a probable contribution of the kinase/phosphatase duo PrkC/PrpC in this phosphorylation event.

## Results and Discussion

### YfnI is specifically phosphorylated *in vitro* by PrkCc on Thr297

**Figure 1:**
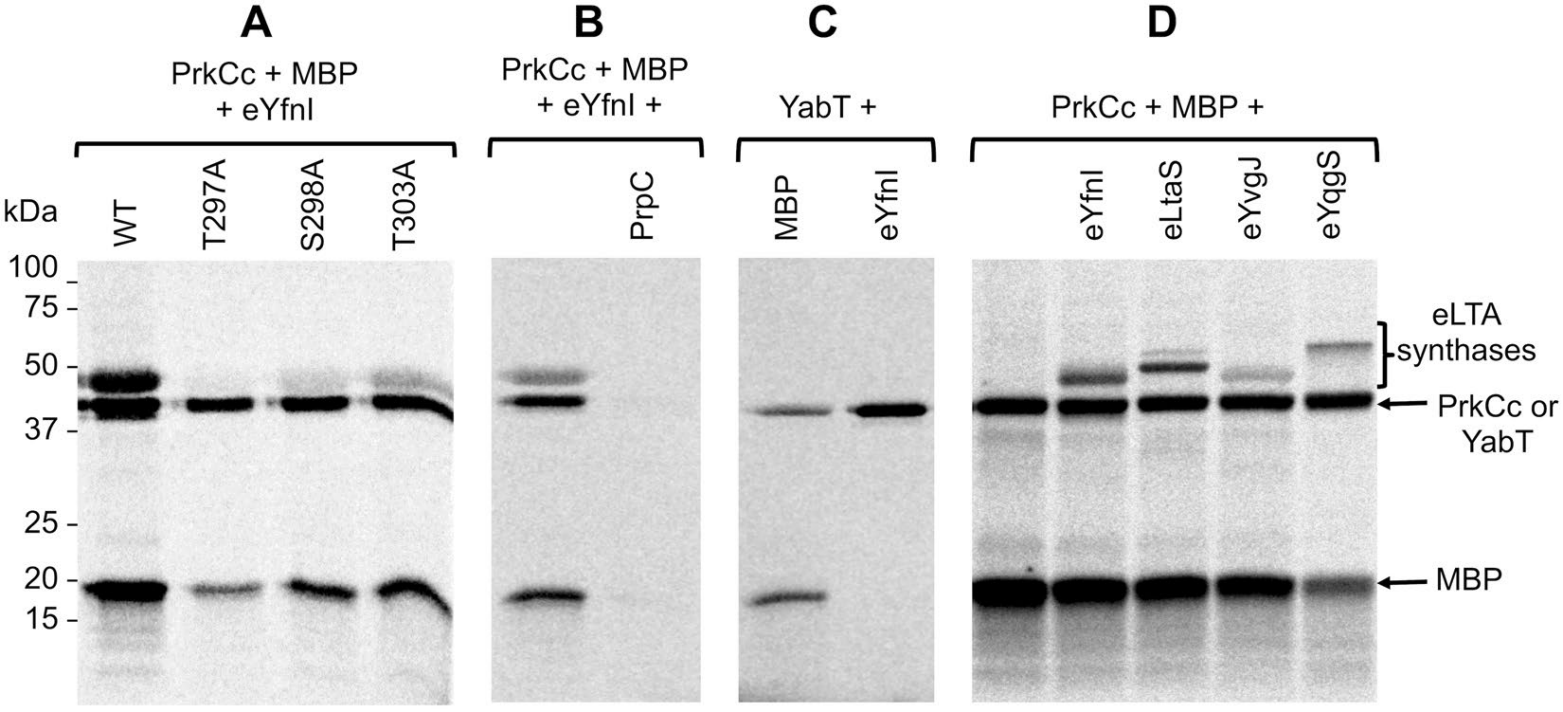
Phosphorylation of eYfnI and the extracellular domains of other LTA synthases by PrkC. (**A**) *In vitro* phosphorylation assays of eYfnI and eYfnI variants by PrkC. The catalytic domain of PrkC, PrkCc, was incubated with [γ-^33^P] ATP, MBP and eYfnI-WT or the eYfnI-T297A, eYfnI-S298A and eYfnI-T303A variants at 37 °C for 15 min. Samples were separated by SDS-PAGE and visualized by autoradiography. The upper bands correspond to the phosphorylated form of eYfnI, the band below to the autophosphorylated PrkCc and the lower band to the phosphorylated MBP. (**B**) *In vitro* phosphorylation of eYfnI by PrkCc and dephosphorylation by PrpC. PrkCc was incubated with [γ-^33^P] ATP, MBP and eYfnI at 37 °C for 10 min (lane 1) then PrpC was added to the reaction and incubated for 10 min at 37 °C (lane 2). (**C**) *In vitro* phosphorylation assays of eYfnI by YabT. The cytoplasmic domain of YabT was incubated with [γ-^33^P] ATP, MBP and eYfnI at 37 °C for 15 min. Samples were separated by SDS-PAGE and visualized by autoradiography. The upper bands correspond to the autophosphorylated YabT and the lower band to MBP-P. (**D**) *In vitro* phosphorylation assays of the extracellular domains of LTA synthases by PrkCc. PrkCc was incubated with [γ-^33^P] ATP, MBP and the extracellular catalytic domain of YfnI, LtaS, YvgJ and YqgS at 37 °C for 15 min. Samples were separated by SDS-PAGE and visualized by autoradiography. The upper bands correspond to the phosphorylated eLTA synthases, the band below to PrkCc-P and the lower band to MBP-P. Full-length autoradiograms are presented in the supplemental data. Coomassie stained gels showing the amount of all purified proteins used in the phosphorylation tests are presented in Fig. S2.

The four *B*. *subtilis* LTA synthases possess a high degree of sequence similarity (Fig. S1). Two of them were found to be phosphorylated in several studies but their phosphorylation site is ambiguous. Analysis of the crystal structure of eLtaS showed a phosphate molecule linked to the Thr297 in its catalytic site^5^ whereas LtaS was shown to be phosphorylated on Ser298 in a *B*. *subtilis* phosphoproteome study^14^. In the same phosphoproteome study, YfnI was found to be phosphorylated on residue Ser298. In contrast, a peptide of YfnI with three potential phosphorylation sites on Thr297, Ser298 and Thr303 has been detected in another phosphoproteome of *B*. *subtilis*^13^. We thus decided to test the phosphorylation of YfnI *in vitro* using different protein variants. The identified residues are located within the extracellular domain of YfnI, we therefore produced and purified eYfnI, eYfnI-T297A, eYfnI-S298A and eYfnI-T303A, and removed the 6His-tag (that also contains several Thr residues). Then we tested their phosphorylation using the purified catalytic domain of the Ser/Thr kinase PrkC (PrkCc) and radioactive ATP (Fig. 1A). The Myelin Basic Protein (MBP) was added in the reaction mix since it stimulates PrkC kinase activity^22^ and serves also as a phosphorylation control by being an exogenous protein kinases substrate^23,24^. We found that eYfnI was indeed phosphorylated by PrkC *in vitro* (lane 1) and that the main phosphorylation site is the Thr297 since all radioactive signal was lost for the eYfnI-T297A variant (lane 2) whereas some residual phosphorylation signal was detected for eYfnI-S298A and eYfnI-T303A (lanes 3 and 4). We also checked if eYfnI-P could be dephosphorylated by PrpC, the phosphatase associated with PrkC and we found that eYfnI-P was indeed dephosphorylated by PrpC (Fig. 1B, lane 2). In order to determine if this phosphorylation was specific to the PrkC kinase, we tested if eYfnI could be phosphorylated *in vitro* by YabT, another Ser/Thr kinase of *B*. *subtilis*, (Fig. 1C). In the same phosphorylation conditions, eYfnI was not phosphorylated by the catalytic domain of YabT (Fig. 1C, lane 2) suggesting that PrkC is able to specifically phosphorylate YfnI on its Thr297.

Since the eLtaS protein was also phosphorylated in the crystal structure on residue Thr297^5^ and considering that all LTA synthases have very similar sequences, particularly in the region around this threonine (Fig. S1), we next tested if the other LTA synthases, eLtaS, eYvgJ and eYqgS, could also be phosphorylated *in vitro* by PrkCc (Fig. 1D). We found that all of them were phosphorylated by PrkCc on their extracellular domain.

In the paper describing the 3D-structure of eLtaS, the authors showed that the replacement of the Thr297 by an Ala residue abolished LtaS function^5^. They proposed that this phosphorylatable Thr297 is probably essential for the production of poly(GroP) and that the presence of a phosphoryl group on the active center of the enzyme would inhibit LtaS activity. They also suggested that Thr297 phosphorylation rather mimics an intermediate state in the formation of poly(GroP) than a phosphorylation by a kinase since this residue is located in a deep pocket^5^. Despite its buried localization in the structure, our results indicate that this Thr297 is accessible for a phosphorylation by the protein kinase PrkCc. Furthermore, since PrkCc was able to phosphorylate eLtaS, eYfnI, eYvgJ and eYqgS *in vitro*, we can consider a possible common regulatory mechanism for the four *B*. *subtilis* LTA synthases.

### YfnI interacts with PrkCc by bacterial two-hybrid

**Figure 2:**
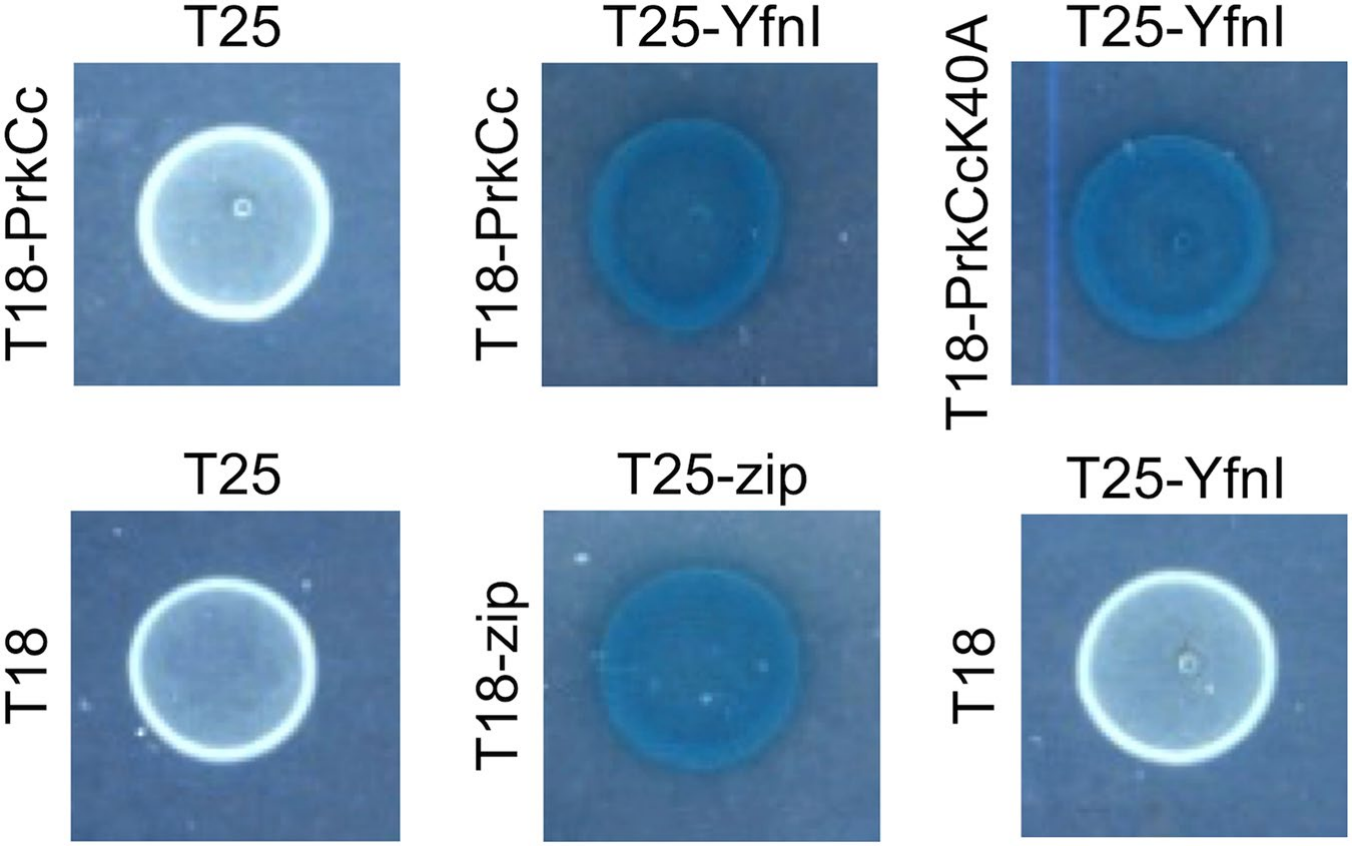
PrkC interacts with YfnI. Interactions detected by bacterial two-hybrid assay. Interactions between the catalytic domain of the kinase, PrkCc (WT or mutated for the catalytic residue K40A) and the entire protein YfnI fused to T18 and T25 domains of the adenylate cyclase respectively are presented. Zip was used as a positive control. BTH101 transformed with the empty vectors T18 and T25, the empty vector T25 and vector T18-PrkCc or the empty vector T18 and vector T25-YfnI used as negative controls.

If LTA synthases are phosphorylated *in vivo* by the *B*. *subtilis* PrkC kinase, it would need to take place in the cytoplasm of the cell before the export of the extracellular domain of the LTA synthases. The folding of the proteins is probably not fully complete allowing access to the future active sites. Consequently, the catalytic domains of the four LTA synthases should interact with that of PrkC. To get further evidence that these interactions might take place in the bacterial cell and that PrkC could phosphorylate YfnI (and eventually the other LTA synthases) *in vivo*, we produced plasmids (Table 1) to test the interaction between YfnI and the catalytic domain of PrkC (PrkCc) or PrkCc mutated for its catalytic residue K40A using a bacterial two-hybrid system (Fig. 2). This analysis indicated an interaction between PrkCc and YfnI *in vivo*, which reinforces the hypothesis that YfnI is a PrkC substrate. As expected, the interaction with YfnI is not lost when PrkCc-K40A is used since this mutation kills the catalytic activity of the kinase but not its ability to interact with its protein substrate.

**Table 1:** *B*. *subtilis* strains and plasmids used in this work.

Strain/plasmid	Relevant characteristics	Source/reference
WT168	*trpC2*	lab collection
1A962	*trpC2 prpC*Δ*1*	^33^
1A963	*trpC2 prkC*Δ*1*	^33^
BKE15760	*trpC2 prpC::erm*	BGSC
BKE07260	*trpC2 yfnI::erm*	BGSC
BKE33360	*trpC2 yvgJ::erm*	BGSC
BKE24840	*trpC2 yqgS::erm*	BGSC
ANG1693	*trpC2 ltaS::kan*	^9^
SG394	*trpC2 yfnI*(*WT*); *kan*	This work
SG397	*trpC2 yfnI*(*T297A*); *kan*	This work
SG399	*trpC2 yfnI*(*T297D*); *kan*	This work
SG445	*trpC2 yfnI::kan*	This work
SG600	*trpC2 ltaS::cat*	This work
SG601	*trpC2 ltaS::cat*; *yfnI::erm*	This work
SG609	*trpC2 ltaS::cat*; *prpC::erm*	This work
SG610	*trpC2 ltaS::cat*; *prpC*Δ*1*; *amyE::gfp-prpC*; *spec*	This work
SG584	*trpC2 ltaS::cat*; *yfnI*(*T297A*); *kan*	This work
SG583	*trpC2 ltaS::cat*; *yfnI*(*T297D*); *kan*	This work
SG577	*trpC2 ltaS::kan*; *prkC*Δ*1*	This work
SG470	*trpC2 yvcK::cat*	^34^
SG533	*trpC2 yvcK::cat*; *prkC*Δ*1*	This work
SG525	*trpC2 yvcK::cat*; *prpC*Δ*1*	This work
SG587	*trpC2 yvcK::cat*; *yfnI::kan*	This work
SG588	*trpC2 yvcK::cat*; *ltaS::kan*	This work
SG579	*trpC2 yvcK::cat*; *yfnI*(*T297A*); *kan*	This work
SG580	*trpC2 yvcK::cat*; *yfnI*(*T297D*); *kan*	This work
SG582	*trpC2 yvcK::cat*; *prkC*Δ*1*; *yfnI*(*T297D*); *kan*	This work
SG586	*trpC2 yvcK::cat*; *prpC*Δ*1*; *yfnI*(*T297A*); *kan*	This work
SG546	*trpC2 yvcK::cat*; *yqgS::erm*	This work
SG544	*trpC2 yvcK::cat*; *yvgJ::erm*	This work
psG1154-*yfnI-FLAG*		This work
psG1154-*yfnI*(*T297A*)*-FLAG*		This work
psG1154-*yfnI*(*T297D*)*-FLAG*		This work
pProEX-*eYfnI*		^9^
pProEX-*eYfnI*(*T297A*)		This work
pProEX-*eYfnI*(*T297D*)		This work
pProEX-*eltaS*		^9^
pProEX-*eyvgJ*		This work
pProEX-*eyqgS*		This work
T18, T18-zip		^35^
T18-*prkCc*		^19^
T18-*yfnI*		This work
T25, T25-Zip		^35^
T25-*yfnI*		This work
T25-*prkCc*		^19^
pBAD-*prkCc*		^22^
pQE30-*yabT*		^25^

### YfnI phosphorylation is not involved in its export to the extracellular medium

**Figure 3:**
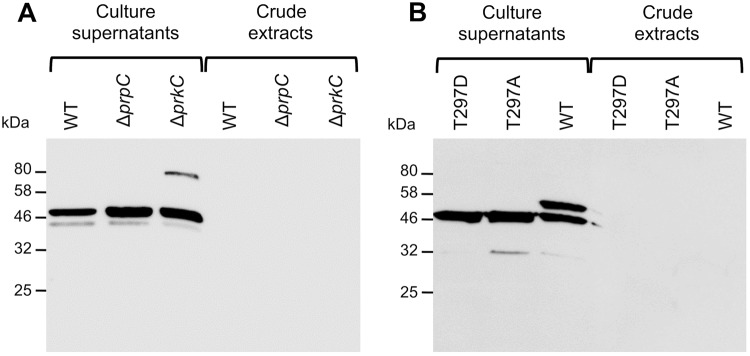
eYfnI export and release are independent of its phosphorylation state. (**A**) Localization of YfnI-FLAG by western blot experiments using anti-FLAG antibodies. Strains expressing *yfnI-flag* in a WT, Δ*prkC* or Δ*prpC* genetic background were grown in LB medium containing 0.5% xylose until the end of exponential phase. Subsequently, protein extracts from culture supernatants or bacterial pellets were prepared and separated by SDS-PAGE and the FLAG-tagged proteins visualized by western blot using anti-FLAG antibodies. The eYfnI-FLAG domain was only detected in the culture supernatants for all three strains tested. (**B**) Determination of the localization of WT, phosphomimetic (T297D) and phosphoablative (T297A) YfnI-FLAG by western blot analysis. Protein extracts from culture supernatants or bacterial pellets from strains grown in LB medium containing 0.5% xylose until the end of exponential phase and expressing *yfnI-T297D-flag*, *yfnI-T297A-flag* or *yfnI-flag* were prepared and separated by SDS-PAGE and the FLAG-tagged proteins detected by western blot using anti-FLAG antibodies. The extracellular domains of the three proteins were only detected in the culture supernatant fractions. Coomassie stained gels showing the amount of proteins in the extracts used in these western blots are presented in Fig. S3. Specificity of anti-FLAG antibodies is shown in Fig. S4.

One issue that is difficult to reconcile is that a protein with an exported domain could be phosphorylated by a kinase that has a cytoplasmic catalytic domain or dephosphorylated by a cytoplasmic phosphatase. This suggests that phosphorylation or dephosphorylation events occur before the export of the extracellular domain of YfnI that is then removed by specific proteolysis and released in the growth medium. The phosphorylation of YfnI could thus have a regulatory effect on the export of its catalytic domain through the cytoplasmic membrane or on its release to the medium in order to control its activity. To test this hypothesis, we constructed *B*. *subtilis* strains producing YfnI fusion proteins with a C-terminal FLAG peptide that allowed us to detect the protein using anti-FLAG antibodies. Antibody specificity was verified on a WT strain 168 that does not produce FLAG tag fused protein (Fig. S4). Site directed mutagenesis was performed on the plasmid carrying *yfnI* fused to the *flag* tag sequence to produce a protein mimicking a phosphorylated protein (YfnI-T297D-FLAG) or an unphosphorylated protein (YfnI-T297A-FLAG) (Table 1). This experimental artifice is commonly used to mimic a protein that is never (replacement of the phosphorylated residue by Ala) or always (replacement by Asp) phosphorylated^25,26^. In addition to being a phosphoablative protein, YfnI-T297A-FLAG is expected to be inactive since the LtaS-T297A protein has been shown to be inactivated by the same mutation^5^. *B*. *subtilis* strains expressing WT or the mutant YfnI variants were grown in LB medium and proteins from the culture supernatant and the bacterial cell pellets analyzed. The fusion proteins were also produced in strains deleted for *prkC* or *prpC* and their localization compared to that in a WT strain. The extracellular domain of YfnI-FLAG proteins could only be detected in the culture supernatant in the three strains analyzed (Fig. 3A). Furthermore, for all three YfnI variants tested (WT, phosphoablative or phosphomimetic), the extracellular domain was released in the culture medium (Fig. 3B). These data suggest that the phosphorylation state of YfnI does not regulate its export or its release by the type I signal peptidases.

### All forms of YfnI bind Glycerol-1-P with similar affinity

In order to analyze the role of this phosphorylation on YfnI properties, we first tested if it affects the affinity of the enzyme for the substrate mimic, GroP. For this purpose, the unphosphorylated protein, a phosphorylated protein preparation (eYfnI was beforehand incubated with PrkCc), and the inactive (eYfnI-T297A) or potential phosphomimetic (eYfnI-T297D) forms of the protein were incubated with GroP then analyzed by isothermal shift assays. Surprisingly, we found that the phosphorylated or modified forms of eYfnI were all able to bind the substrate with only a slightly smaller affinity (Table 2) than the unphosphorylated WT form (apparent K_D_ = 2.9 +/− 0.2 mM). This result indicates that there is enough room in the catalytic site to receive the GroP molecule even when the Thr297 is phosphorylated or replaced by another residue. Recently, the structure of eLtaS from *Listeria monocytogenes* has been solved^27^ showing a phosphorylated catalytic threonine (Thr307_Lm_ = Thr297_Bs_). Although the authors suggest that this phosphorylation is not physiological, they characterized two functional GroP binding sites in the protein that allows the simultaneous occupation of the phosphorylation of Thr307 and a GroP molecule in each of the sites within a compatible distance to permit the enzymatic catalysis. This second binding pocket has been identified in eLtaS from *B*. *subtilis* as well but a significant conformational change has to occur before the catalytic reaction^27^. Furthermore, LTA synthases belong to the alkaline phosphatase and arylsulfatase superfamily for which Ser and Thr residues are often phosphorylated to be active^28^ suggesting that a similar mechanism may be necessary for LtaS and for the other LTA synthases in *B*. *subtilis*.

**Table 2:** Binding affinity for GroP.

Protein	Apparent K_D_ (mM)
YfnI	2.9 +/− 0.2
YfnI + P-YfnI	9.2 +/− 0.5
YfnI-T297A	8.0 +/− 0.3
YfnI-T297D	5.4 +/− 0.3

### The phosphomimetic form of YfnI is active *in vivo* to synthesize LTAs

**Figure 4:**
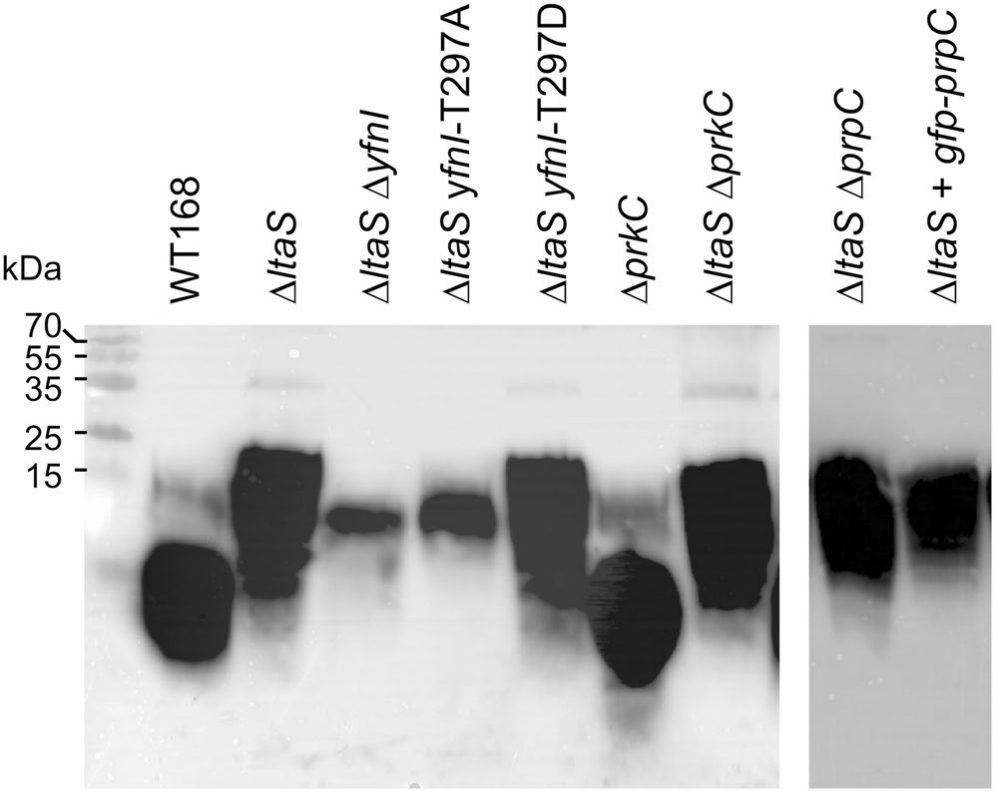
Characterization of LTA production and YfnI activity *in vivo*. LTA detection by western blot. The different *B*. *subtilis* strains were grown at 37 °C in CE minimum medium supplemented with 0.5% gluconate for 6 hours. 1 ml aliquots were removed, samples prepared and analyzed by western blot using anti-LTA antibodies as previously described^9^. Sizes of protein standards run in parallel are shown in the left of the panel. The figure is made by grouping two independent cropped blots treated in the same conditions. Full-length blots are presented in the supplemental data.

In order to test the activity of YfnI *in vivo*, we took advantage that the LTA chains are longer when produced by YfnI instead of LtaS and constructed strains in the Δ*ltaS* background in which YfnI-WT, YnfI-T297A or YfnI-T297D were produced, as well as positive and negative control strains in which the genes coding for PrkC or PrpC were deleted or overexpressed as described in the Methods section (Table 1). The strain overexpressing GFP-PrpC was used as a negative control; in this strain all PrpC protein substrates are supposed to be dephosphorylated. The activity of the GFP-PrpC fusion protein was previously checked as shown in the Fig. S5. We then performed western blot experiments using anti-LTA antibodies as described in^9^ to determine the composition of LTA polymers in these strains and estimate the activity of the YfnI protein (Fig. 4). We observed that the LTA mobility on the gel was similar to that of the Δ*ltaS* strain when the phosphomimetic form of YfnI, YfnI-T297D, was produced whereas the LTA composition was similar to that of an Δ*ltaS* Δ*yfnI* double mutant strain when the phosphoablative form of YfnI, YfnI-T297A, was produced. These results show that, as expected, the replacement of Thr297 by Ala inactivated the YfnI enzyme. In contrast, the replacement by Asp had no effect on its enzymatic activity and YfnI-T297D was able to synthesize LTAs *in vivo* similar to the WT protein. However, it was proposed that this Thr297 may be essential for the production of poly(GroP) and the presence of a phosphoryl group on the active center of the enzyme would inhibit LtaS activity^5^. In a Δ*ltaS* strain, we observed that neither the deletion of *prpC* or *prkC*, nor the overproduction of GFP-PrpC had an effect on the synthesis of the longer LTA chains (Fig. 4) since the mobility of LTA was similar to that of the Δ*ltaS* strain. So, even if the PrkC and PrpC kinase/phosphatase duo is able to phosphorylate/dephosphorylate YfnI *in vitro*, we were unable to determine by this approach, an involvement of these enzymes in the phosphorylation and dephosphorylation of YfnI *in vivo*. We concluded that, if YfnI phosphorylation is physiological, this suggests that either PrkC faintly phosphorylates YfnI or/and the effect of this phosphorylation on its activity is weak within the cell.

### Only two of the four LTA synthases are able to complement a Δ*yvcK* mutant

**Figure 5:**
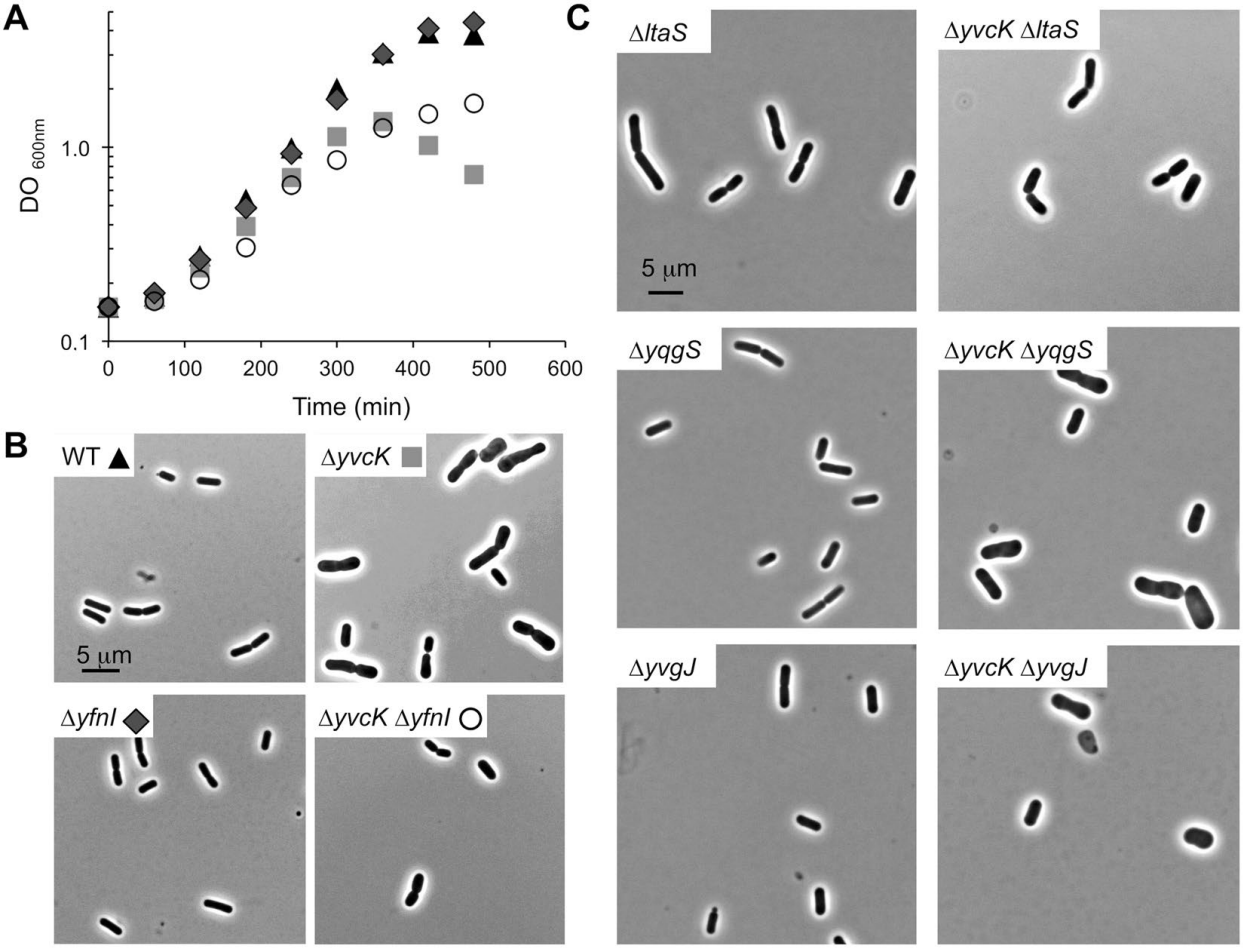
Analysis of the growth and cell morphology of a Δ*yvcK* mutant following the deletion of genes coding for LTA synthases. (**A**) Growth curves. *B*. *subtilis* strains WT168 (black triangle), Δ*yvcK* (grey square), Δ*yfnI* (dark grey diamond) and Δ*yvcK* Δ*yfnI* (white circle) were grown at 37 °C in CE minimum medium supplemented with 0.5% gluconate and OD_600_ was measured hourly. (**B**) Morphology analysis. Images of strains described in (**A**) were taken after 6 hours of growth using a Zeiss Upright Axio Imager M2 microscope. (**C**) Morphology analysis of other LTA synthases mutant strains. The strains Δ*ltaS*, Δ*yvcK* Δ*ltaS*, Δ*yqgS*, Δ*yvcK* Δ*yqgS*, Δ*yvgJ* and Δ*yvcK* Δ*yvgJ* were grown at 37 °C in CE minimal medium supplemented with 0.5% gluconate and microscopy images were taken after 6 hours of growth.

To analyze the possible participation of PrkC and PrpC in the regulation of YfnI activity *in vivo*, we then decided to evaluate the effect of YfnI phosphorylation with another method. In a previous transposon mutagenesis screen looking for Δ*yvcK* suppressive mutations^21^, a *yfnI* mutant has been identified as suppressor strain. That is, insertion of a transposon in *yfnI* restored the growth of the Δ*yvcK* mutant in the presence of neoglucogenic carbon sources. We thus used this characteristic to test the activity of YfnI in *B*. *subtilis* cells. The four strains WT, Δ*yvcK*, Δ*yfnI* and Δ*yvcK* Δ*yfnI* were grown in liquid CE minimum medium supplemented with 0.5% gluconate and we confirmed that the deletion of *yfnI* in a Δ*yvcK* strain was able to partially restore the growth of the *yvcK* mutant strain (Fig. 5A). We also checked the cell morphology after 6 hours of growth since it has been described that the Δ*yvcK* mutant forms at this time point cells with abnormal shapes which eventually lyse (Fig. 5B). The single mutant Δ*yfnI* used as negative control had the same shape as a WT strain. Again, we observed that the deletion of *yfnI* in a Δ*yvcK* background strain restored normal cell morphology. We then wondered if this would be true following the deletion of the genes coding for the other LTA synthases in *B*. *subtilis* and constructed the double deletion strains Δ*yvcK* Δ*ltaS*, Δ*yvcK* Δ*yvgJ* and Δ*yvcK* Δ*yqgS* and tested them with the same morphological screen (Fig. 5C). All single mutant (Δ*ltaS*, Δ*yvgJ* and Δ*yqgS*) strains had a WT shape as expected. We found that only the absence of *ltaS* or *yfnI* was able to complement the morphological phenotype of the Δ*yvcK* mutant whereas the absence of one of the other two enzymes, YvgJ or YqgS, had no effect. These results confirm that the activities of the LTA synthases *in vivo* are not perfectly redundant, consistent with previously described results^5,9^.

### The phosphomimetic form of YfnI is active *in vivo* and unable to complement a Δ*yvcK* mutant

**Figure 6:**
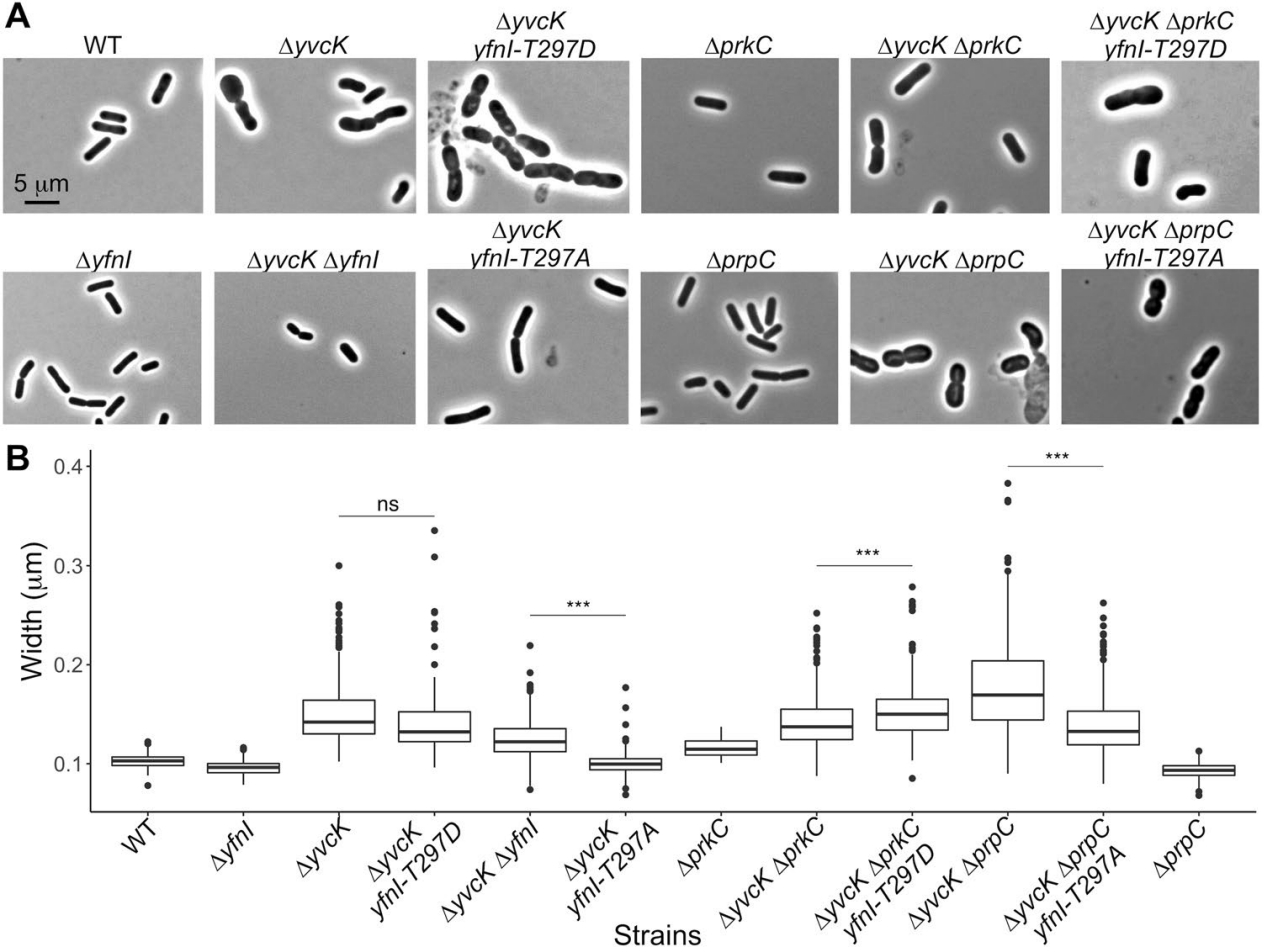
Morphology and cell width determination for Δ*yvcK* mutant strains with modified YfnI activities. (**A**) Morphology analysis of strains affected for YfnI phosphorylation. Strains WT168, Δ*yvcK*, Δ*yfnI*, Δ*yvcK* Δ*yfnI* Δ*yvcK yfnI-T297A*, Δ*yvcK yfnI-T297D*, Δ*yvcK* Δ*prkC*, Δ*yvcK* Δ*prkC yfnI-T297D*, Δ*yvcK* Δ*prpC* and Δ*yvcK ∆prpC yfnI-T297A* were grown at 37 °C in CE minimum medium supplemented with 0.5% gluconate and microscopy images were taken after 6 hours of growth. (**B**) Cell width measurement. The boxplot shows the average width of about 150 bacteria measured from images of each strain presented in panel A using the ImageJ software. P-values were calculated with a Wilcoxon test. When two strains were compared, ns means not significant and ***means p < 0.001.

In order to test the relevance of YfnI phosphorylation *in vivo*, we then used the same morphological screen to test the activity of phosphomimetic and phosphoablative forms of YfnI and for this, constructed strains expressing *yfnI-T297A* or *yfnI-T297D* in a Δ*yvcK* genetic background (Table 1 and Fig. 6A). Firstly, we observed that the bacteria producing the inactive phosphoablative form of YfnI (YfnI-T297A) had, as expected, a normal shape similar to the double mutant strain. By contrast, the cells generating the phosphomimetic form of YfnI had a shape similar to the cells of the Δ*yvcK* mutant suggesting that YfnI-T297D is active *in vivo*. These results are in agreement with the above described data and highlight that YfnI-T297D is active whereas YfnI-T297A is inactive (Fig. 4). We next analyzed the morphology of cells from the Δ*yvcK* Δ*prkC* and Δ*yvcK* Δ*prpC* deletion strains after growth for 6 hours in liquid CE minimum medium with 0.5% gluconate (Fig. 6A). We observed that cells lacking the PrpC phosphatase had an abnormal shape; this suggests that YfnI is active in this strain. In the absence of the phosphatase, YfnI could potentially be locked in its phosphorylated form. These results are consistent with the data obtained with YfnI-T297D. Cells lacking the PrkC kinase were wider than the WT or Δ*yvcK* Δ*yfnI* cells but they did not show a shape defect similar to a Δ*yvcK* mutant. In this strain, if PrkC is the kinase responsible of YfnI phosphorylation, we expected that YfnI would be in an unphosphorylated state and therefore not fully active which should lead to the return to a normal cell shape. However, the results suggest that another kinase could replace PrkC *in vivo* to phosphorylate YfnI in a Δ*prkC* mutant and/or the unphosphorylated form has activity: in this case, the phosphorylation of Thr297 acts as a booster. However, there might be a difference *in vivo* between the replacement of Thr297 by Ala which completely inhibits the activity of YfnI and the suppression of the kinase which leads to the production of YfnI in the unphosphorylated state that might still be partially active. In order to address this question, we constructed two additional strains in which YfnI-T297A was produced in a Δ*yvcK* Δ*prpC* background or where YfnI-T297D was produced in a Δ*yvcK* Δ*prkC* background (Fig. 6A). As expected, even if the complementation was not complete, the morphology defects appeared to be somewhat reduced for the Δ*yvcK* Δ*prpC yfnI-T297A* strain and increased for the Δ*yvcK* Δ*prkC yfnI-T297D* strain. To better estimate the effect on cell morphology, we also measured the width of cells from the different strains used in this study (Fig. 6B). The WT *B*. *subtilis* strain 168 and the single mutants Δ*prpC*, Δ*prkC*, and Δ*yfnI* were used as negative controls and showed widths of 0.102, 0.093, 0.116 and 0.096 µm respectively (median width of 0.105 µm) whereas the single mutant Δ*yvcK*, used as positive control, presented a width of 0.150 µm. The cell width data collected were consistent with the shape of bacteria on the images but also showed that cells of the single Δ*prkC* mutant were already a bit wider (0.116 µm) than WT cells (0.102 µm), which could explain why the cell shape of the Δ*yvcK* Δ*prkC* deletion strain was not completely restored. Furthermore, the size of the cells was significantly increased for strain Δ*yvcK* Δ*prkC yfnI-T297D* (0.152 µm) compared to strain Δ*yvcK* Δ*prkC* (0.140 µm) and reduced for the strain Δ*yvcK* Δ*prpC yfnI-T297A* (0.138 µm) compared to strain Δ*yvcK* Δ*prpC* (0.171 µm) (Fig. 6B).

In conclusion, these results suggest that there might be a regulation of YfnI activity *in vivo* through its phosphorylation state that can be at least partially mediated by the PrkC/ PrpC kinase and phosphatase proteins. It is also important to remember that PrkC and PrpC have multiple substrates in the cell involved in several cellular processes. Therefore, deletion of *prkC* and *prpC* may have quite pleotropic effects explaining the small differences in cell size observed here. However, our results suggest that PrkC could phosphorylate YfnI *in vivo* but it may be not the only kinase responsible for this phosphorylation event.

## Methods

### Plasmids and strains constructions

Standard procedures for molecular cloning and cell transformation of *B*. *subtilis* and *E*. *coli* were used. All the strains and plasmids used in this study are listed in Table 1. Some strains have been received from the Bacillus Genetic Stock Center (BGSC). Primer sequences used in this study are available upon request. Sequencing of PCR-derived DNA fragments in the plasmid constructs was carried out by GATC-Biotech to ensure error-free amplification.

For the generation of FLAG-fusion proteins, *yfnI* was amplified by PCR using specific primers and inserted between *Apa*I and *EcoR*I sites in the pSG1154 plasmid where the *gfp* gene has been replaced by a sequence encoding a FLAG peptide.

Site directed mutagenesis was then carried out on the resulting plasmid pSG1154-*yfnI*-*flag* to generate fusion proteins with phosphoablative (T297A) or phosphomimetic (T297D) replacements; as well as on plasmid pProEX-e*yfnI* to produce N-terminal 6His-tagged extracellular domains of YfnI carrying the mutations S298A, T303A and the phosphoablative (T297A) or phosphomimetic (T297D) replacements.

To produce the extracellular domains of YvgJ and YqgS with an N-terminal 6His-tag, e*yvgJ* and e*yqgS* were amplified by PCR using specific primers and used to replace e*yfnI* in the pProEX-e*yfnI* plasmid between the BamH*I* and Xba*I* sites.

The flanking sequences of *yfnI* (*yetO*, and *yfnHG* regions) or *ltaS* (*nagB*, and *yflDA* regions) were amplified from chromosomal DNA by PCR with two pairs of primers containing overlapping regions. The two above described fragments and a fragment containing the kanamycin or the chloramphenicol resistance genes respectively were then joined by isothermal assembly using the Gibson Assembly® Master Mix from NEW ENGLAND BioLabs to generate the whole sequences *yetO-kan-yfnHG and nagB-cat-yflDA* and the resulting products were used to transform the WT168 strain to generate the strains SG445 and SG600 (Table 1).

To generate chromosomal mutations, the pSG1154-*yfnI*-*flag* and derived plasmids were used to amplify the modified *yfnI* genes. The flanking sequences of *yfnI* (*yetO* and *yfnHG* genes) were amplified from the chromosomal DNA by PCR with two pairs of primers containing overlapping regions. A global PCR using the three above described fragments and a fragment containing the kanamycin resistance gene were then used to amplify the whole sequence *yetO-yfnI* (*WT or T297A or T297D*)*-kan-yfnHG* and the resulting DNA were used to transform the BKE07260 strain to generate the strains SG394, SG399 and SG397 (Table 1).

For the generation of fusion proteins for the adenylate cyclase-based two-hybrid assay, the DNA encoding YfnI was amplified by PCR using specific primers and inserted between *Pst*I and *BamH*I sites in the pT25 plasmid as described previously^19^.

### Protein purification

pProEX-derived plasmids allowing the overexpression of N-terminal 6His-tagged extracellular domain of the LTA synthases were used to transform *E*. *coli* Rosetta. Purification of 6His-tagged recombinant proteins was performed with Ni^2+^-NTA resin (Qiagen) as previously described^29^ for eYfnI, eYfnI-T297A, eYfnI-T297D, eYfnI-S298A, eYfnI-T303A, eLtaS, eYvgJ and eYqgS. The tag was then removed by proteolytic digestion with the acTEV protease (10 units for 500 µg of protein during 90 min at 30 °C) and separated from the 6His-tag by a second purification step on Ni^2+^-NTA resin from which the proteins were obtained in the first wash step.

Plasmids allowing the production of 6His-tagged catalytic domains of PrkC and YabT (Table 1) were used to transform *E*. *coli* C41(DE3). Purification of 6His-tagged recombinant proteins was performed with Ni^2+^-NTA resin (Qiagen) as previously described in^22^ and^30^ respectively.

### Protein phosphorylation

2 μg of eYfnI (or extracellular domains of the others LTA synthases) were incubated for 15 min at 37 °C with 2 μg of the catalytic domain of PrkC protein, PrkCc, (or YabT) in a 15 μl reaction mixture containing 10 mM HEPES, pH 8.0, 1 mM MgCl_2_, 2 μg of MBP (Myelin Basic Protein) and 1 mM [γ-^33^P] ATP (1 μCi). MBP stimulates PrkC kinase activity and serves also as a phosphorylation control by being an exogenous protein kinases substrate^22–24^. For the dephosphorylation test of eYfnI, 2 μg of eYfnI were first phosphorylated as described above for 10 min at 37 °C (positive control) and when indicated 1 μg of PrpC was added and incubated for 10 additional minutes. The phosphorylation reaction was stopped by adding 5X SDS-sample buffer to the reaction mixtures before SDS-PAGE analysis. Gels were then dried and exposed to autoradiography.

### Thermal shift assay

In thin-walled 96-well PCR plates, each well (20 μl) contained 10 μM of eYfnI protein (or eYfnI mutated proteins) and 2 μl of the fluorescent SYPRO^®^ Orange dye solution (Molecular Probes, 5000x, diluted to 100x in water), in 40 mM NaCl, 1 mM MgCl_2_, 10 mM Tris/HCl, pH 8.0 and increasing concentrations of GroP (from 0 to 20 mM) and was heated from 25 °C to 65 °C in 0.5 °C steps. The fluorescence intensity (Ex/Em = 470/570 nm) of SYPRO^®^ Orange was monitored using a real-time PCR apparatus CFX96 (Bio-Rad). The fluorescence of SYPRO^®^ Orange dye changes when it interacts with the protein undergoing thermal unfolding. The denaturation temperature (T_m_) was analyzed from the melt peak using CFX Manager software (Bio-Rad). The shift of T_m_ (ΔT_m_) induced by the presence of ligand was plotted against the concentration of ligand. Curve fitting was performed by using Microcal Origin 5.0 software with the Hill equation. To test the affinity of GroP for the phosphorylated form of eYfnI, 400 µg of the protein was first incubated with 50 µg of PrkCc in 10 mM HEPES, pH 8.0, 5 mM MgCl_2_ and 5 mM ATP for 60 min at 37 °C. eYfnI-P was then used as described above.

### Growth tests

*B*. *subtilis* strains were grown in LB or CE minimal medium supplemented with 0.5% gluconate as previously described in^21^.

### Western Blot

For LTA detection, cells were grown at 37 °C in 30 ml of CE minimal medium supplemented with 0.5% gluconate for 6 hours then treated as previously described^9^.

For YfnI-FLAG detection, cells were grown at 37 °C in 30 ml of LB medium containing 0.5% xylose until OD_600_ = 1.2, then centrifuged for 10 min at 4000 rpm at 4 °C. Proteins from cell culture supernatants (SN) were precipitated by addition of 1/10^th^ volume of TCA and incubated for 30 min at 4 °C. SN were centrifuged for 30 min at 4000 rpm and 4 °C then protein pellets were washed twice with acetone, left 5 min to dry then resuspended in 1/100^th^ volume of Laemmli buffer. Crude extracts from cell pellets were obtained by resuspension in 1/30^th^ volume of lysis buffer containing 10 mM Tris-HCl pH 8.0, 150 mM NaCl, 0.1% NP40, 1 mM PMSF, 1 mM DTT, 25 U.ml^−1^ benzonase and 10 mg/ml lysozyme, and incubated for 30 min at 37 °C. 1/10^th^ volume of 10% SDS and 1/2 volume of (2×) Laemmli buffer were added to the extracts. All protein extracts were heated at 100 °C for 10 min. Samples were run on a 12.5% SDS-PAGE and transferred to hybond-ECL membrane by electroblotting. The membrane was blocked with PBS solution containing 5% milk powder (w/v), for 3 hours at room temperature with shaking, then incubated with anti-FLAG antibodies diluted to 1/1000^th^ overnight at 4 °C. After three washes, the secondary antibody, a peroxidase-conjugated Goat anti-Rabbit (Thermo scientific) antibody was used at 1/2000^th^ dilution for one hour. After three washes, the membrane was incubated with ECL reagents (PerkinElmer) and scanned for chemiluminescence with an ImageQuant LAS4000 (GE Healthcare).

### Microscopy

Strains were grown for 6 h in CE minimal medium supplemented with 0.5% gluconate at 37 °C. Images were obtained by microscopy on a Zeiss Upright Axio Imager M2 microscope. Cell width was measured with the ObjectJ plugin of ImageJ software and a statistical analysis of these data was made using a Wilcoxon test in order to calculate p-values.

### Bacterial two-hybrid assay

The bacterial two-hybrid assay was performed as described in^31,32^. The N-termini of YfnI, PrkCc and PrkCc-K40A were fused to the T18 or T25 catalytic domains of adenylate cyclase as described in^19^ and above. Co-transformed strains of *E*. *coli* BTH101 expressing several combinations of T18 and T25 vectors were plated on LB agar and incubated at 30 °C for 48 hours. One milliliter of LB medium, supplemented with 100 µg/ml of ampicillin, 50 µg/ml of chloramphenicol, and 0.5 mM of IPTG, was inoculated and incubated at 30 °C overnight. Ten microliters of the overnight culture were spotted on the LB medium plates containing appropriate antibiotics, 0.5 mM IPTG and 40 µg/ml Xgal. The plates were incubated at 30 °C overnight.

## Electronic supplementary material

Supplementary information

## Data Availability

Data and materials will be made available upon request.
