# Supplementary material for: Investigation of the phosphorylation of Bacillus subtilis LTA synthases by the serine/threonine kinase PrkC
Source: Sci Rep. 2018 Nov 26;8:17344. doi: 10.1038/s41598-018-35696-7 (PMC6255753; doi:10.1038/s41598-018-35696-7)

Investigation of the phosphorylation of *Bacillus subtilis* LTA synthases by the serine/threonine kinase PrkC

Frédérique Pompeo, Jeanine Rismondo, Angelika Gründling and Anne Galinier

Supplemental Information:

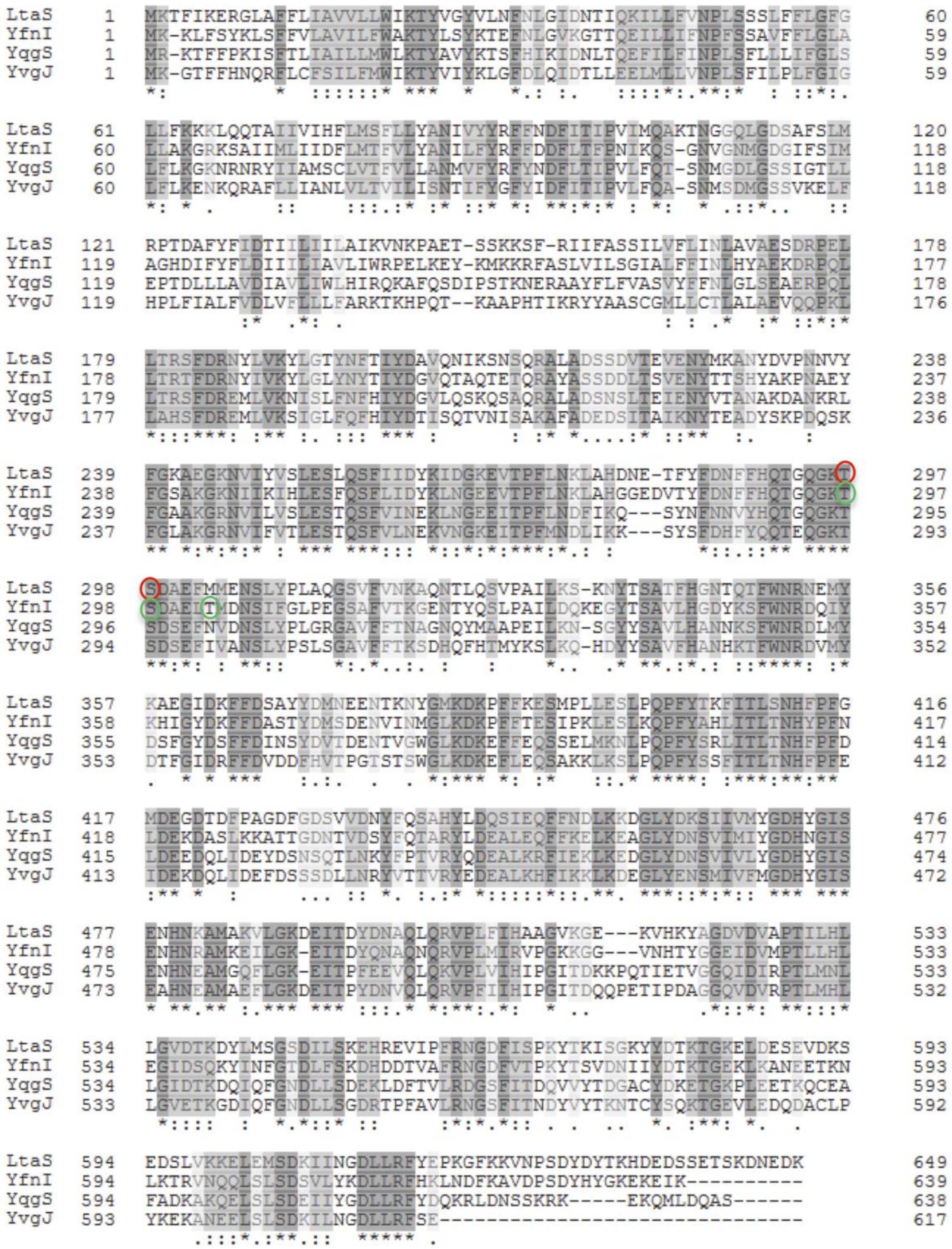

Figure S1. Alignment of the four LTA synthase protein sequences.

The alignment was performed with the Clustal Omega software accessible from UniProt database [1]. The potential phosphorylation sites are circled in red for LtaS and in green for YfnI.

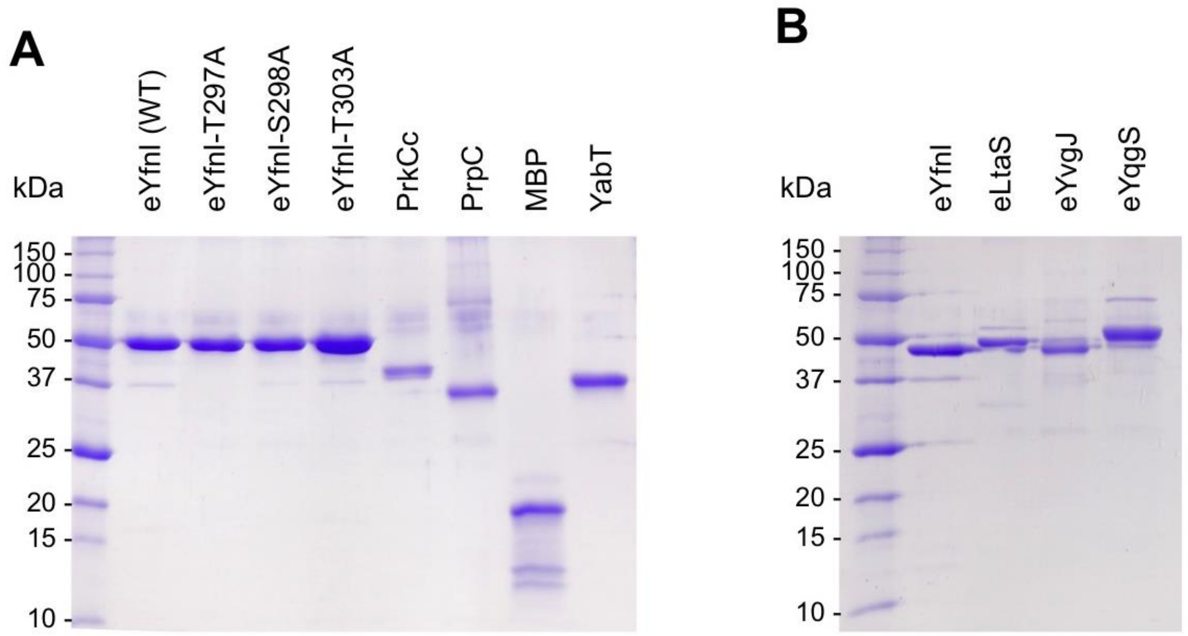

Figure S2. Loading controls for proteins used in the phosphorylation test.

The amount of each protein used in the phosphorylation test described in Figure 1 have been loaded (2 µg except for PrpC = 1 µg) and separated on SDS-PAGE gels then submitted to Coomassie blue staining. **A.** eYfnI (WT), eYfnI-T297A, eYfnI-S298A, eYfnI-T303A, PrkCc, PrpC, MBP, YabT. **B.** eYfnI, eLtaS, eYvgJ, eYqgS.

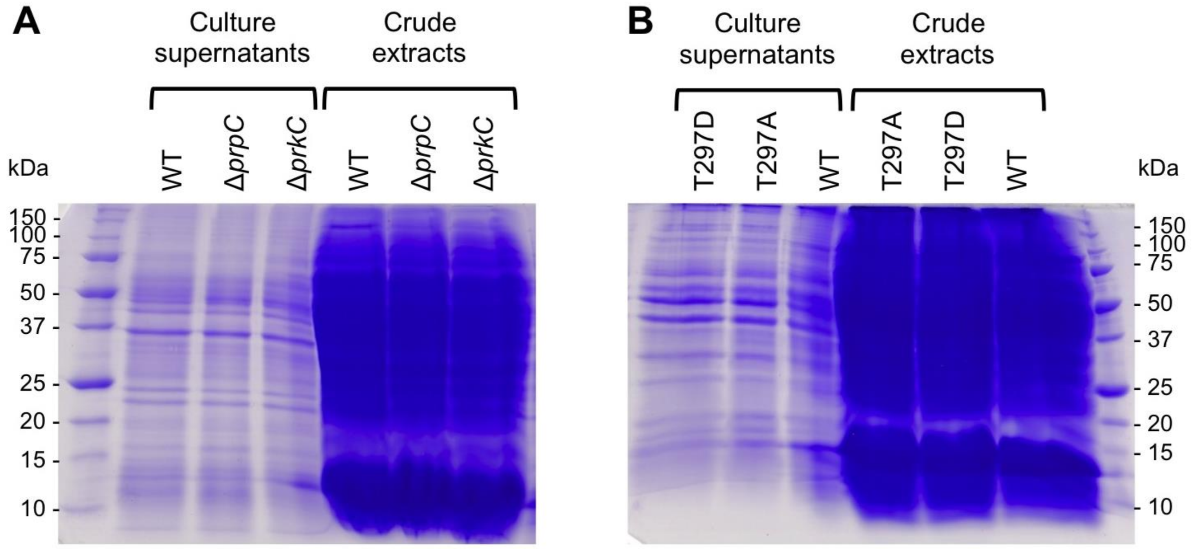

Figure S3. Loading controls for protein extracts used in the localization test.

Equivalent amounts of proteins from culture supernatants and crude extracts used in Figure 3 have been loaded and separated on SDS-PAGE gels then submitted to Coomassie blue staining as loading controls. **A.** Culture supernatants from strains producing YfnI-FLAG in a WT, Δ*prpC* or Δ*prkC* background and crude extracts from strains producing YfnI-FLAG in a WT, Δ*prpC* or Δ*prkC* background. **B.** Culture supernatants from strains producing YfnI-T297D-FLAG, YfnI-T297A-FLAG and YfnI-FLAG and crude extracts from strains producing YfnI-T297D-FLAG, YfnI-T297A-FLAG and YfnI-FLAG.

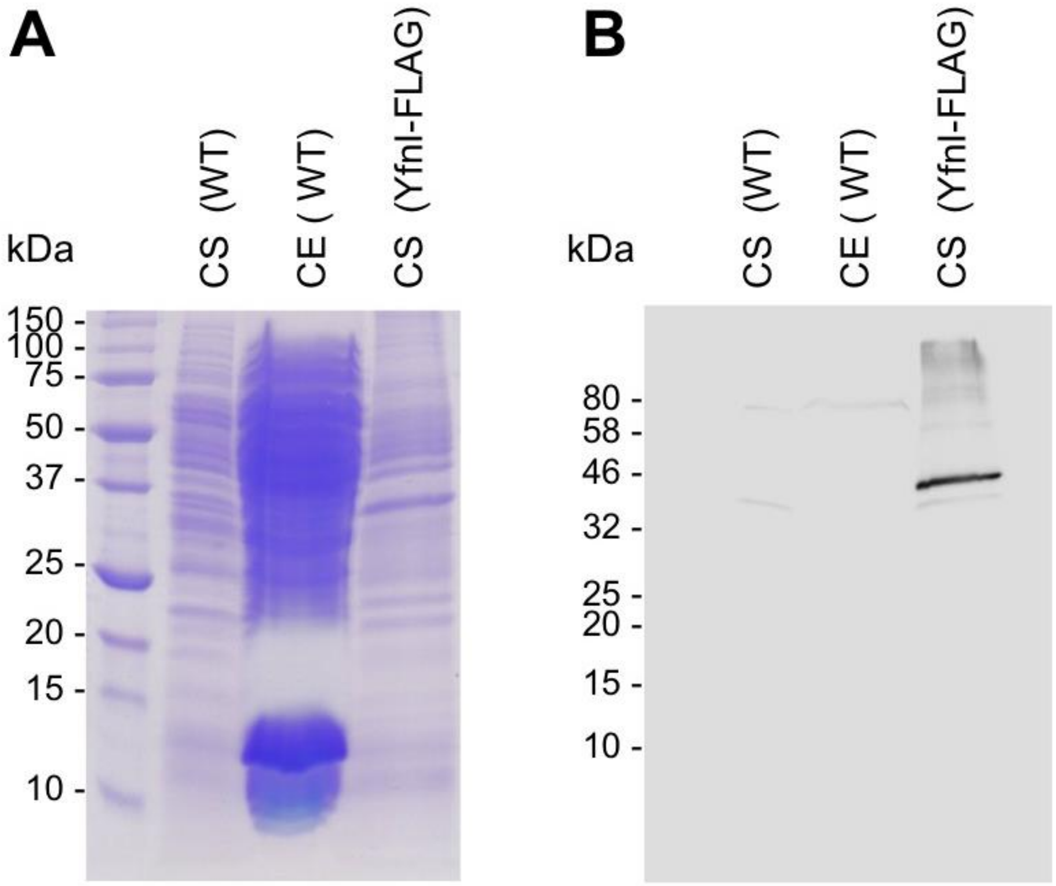

Figure S4. YfnI-FLAG is phosphorylated by PrkCc.

A. Coomassie stained gel showing the amount of proteins in culture supernatants (CS) and crude extracts (CE) from strain WT168 used as negative control to test the specificity of anti-FLAG antibodies and in culture supernatant from strain SG550 producing YfnI-FLAG used as positive control. **B.** Western blot using anti-FLAG antibodies on the same extracts as loaded in A.

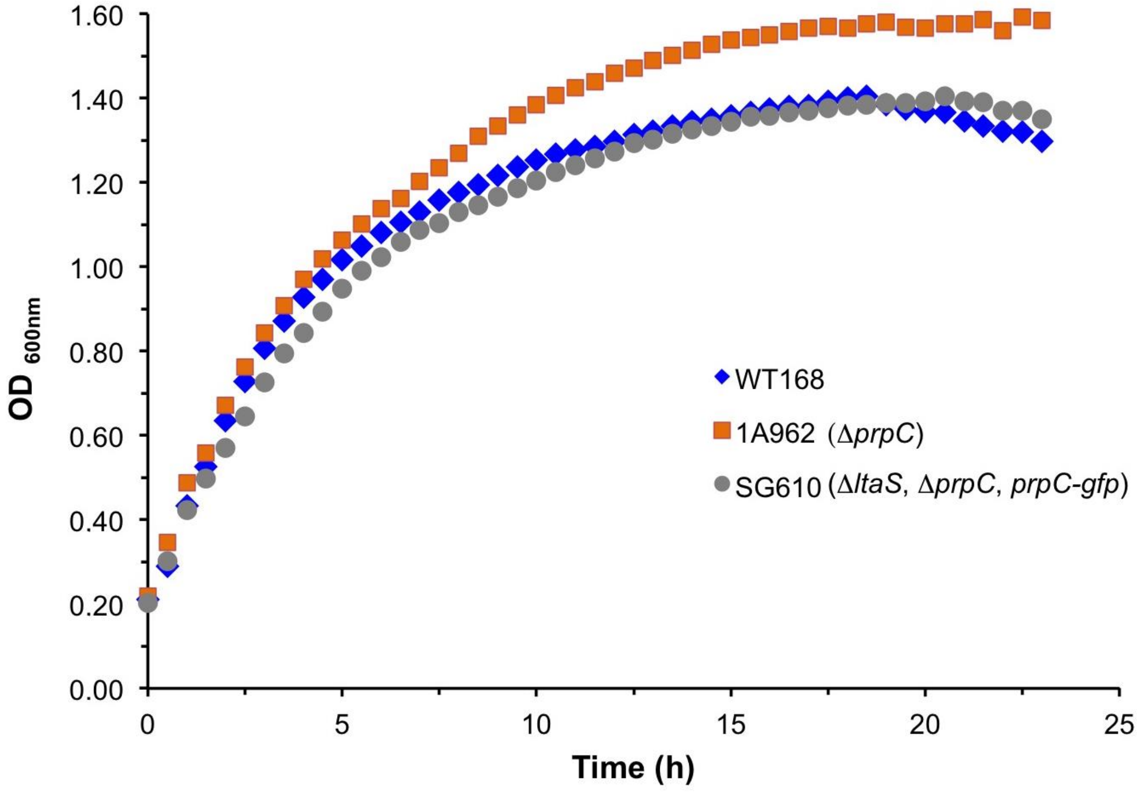

Figure S5. The GFP-PrpC fusion protein is active *in vivo*.

Growth curves of *B. subtilis* strains WT168, 1A962 (Δ*prpC*) and SG610 (Δ*ltaS*, Δ*prpC*, *prpC*-*gfp*) grown at 37°C in LB medium supplemented with 0.5% xylose. OD_{600} was measured hourly.

Additional reference:

1 Sievers, F. *et al.* Fast, scalable generation of high-quality protein multiple sequence alignments using Clustal Omega. *Mol Syst Biol* **7**, 539, doi:10.1038/msb.2011.75 (2011).

Original autoradiograms and blots. Parts used for the manuscript are in the red squares.

Figure 1
Autoradiograms used to construct

- Fig 1A

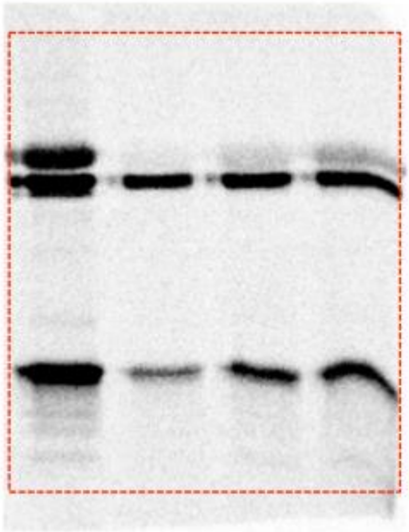

- Fig 1B

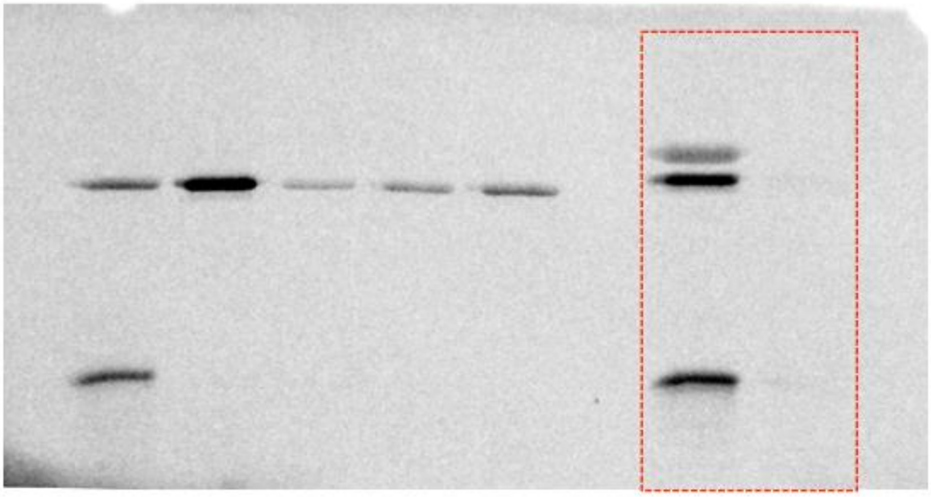

- Fig 1C

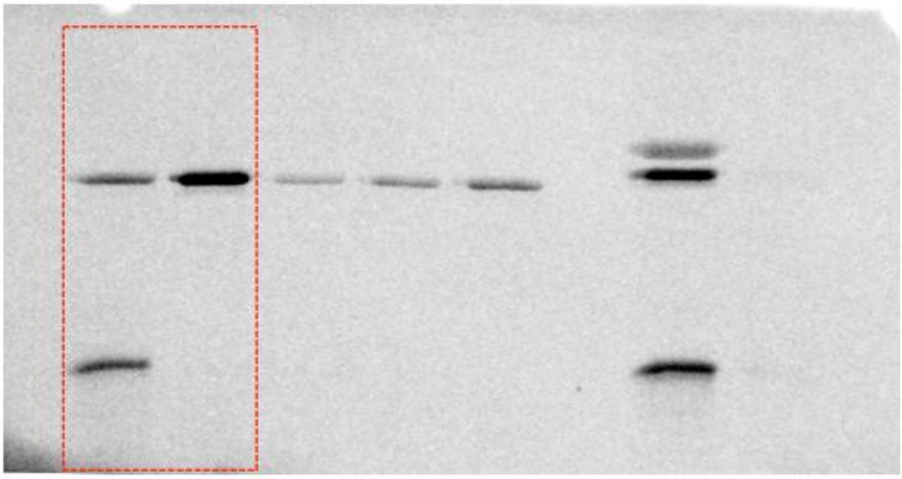

- Fig 1D

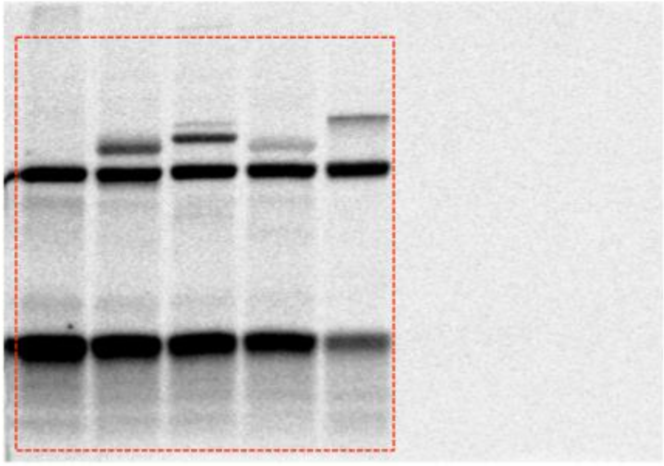

Figure 3
Blots used to construct Fig 3A and Fig 3B

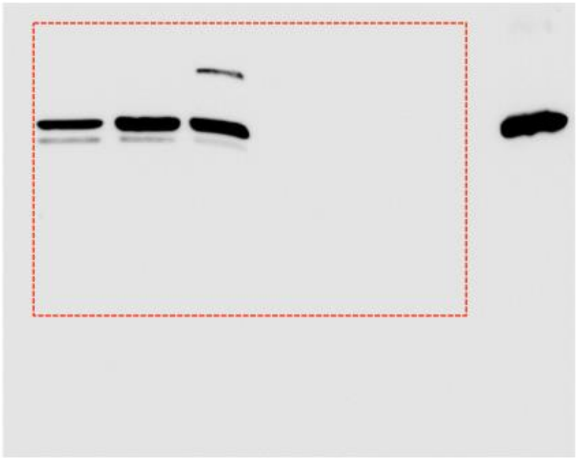

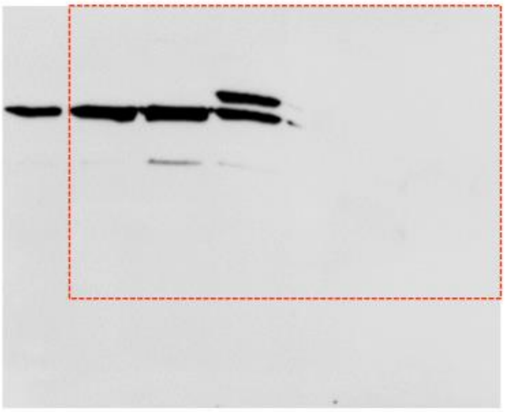

Figure 4
Blots used to construct Fig.4

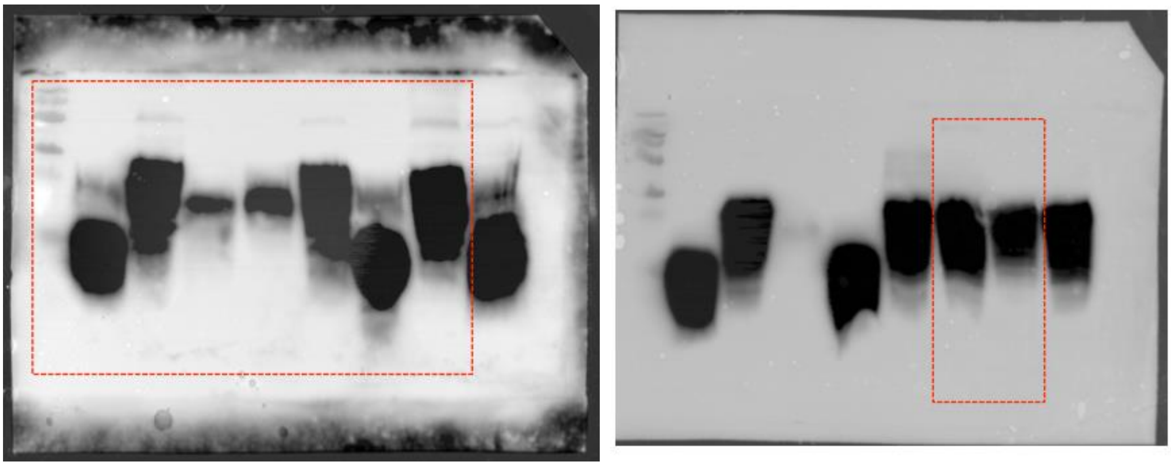

Supplement: Supplementary file 1 — Supplementary information [file 41598_2018_35696_MOESM1_ESM.pdf]
